# Independent Associations of Food Insecurity and Neighborhood Deprivation: Independent Associations with Incident Metabolic Dysfunction–Associated Steatotic Liver Disease in a Diverse U.S. Cohort

**DOI:** 10.3390/nu18162741

**Published:** 2026-08-21

**Authors:** Yong Eun, Marie S. Thearle, Joon Hee Kim, Rhonda K. Trousdale, Joan Culpepper-Morgan

**Affiliations:** 1 Department of Medicine, NYC Health + Hospitals/Harlem, New York, NY 10037, USA; thearlem@nychhc.org; 2 Department of Medicine, Columbia University Vagelos College of Physicians and Surgeons, New York, NY 10032, USA; rhonda.trousdale@nychhc.org (R.K.T.); joan.culpepper-morgan@nychhc.org (J.C.-M.); 3 Independent Researcher, New York, NY 10075, USA; jh.junikim@gmail.com; 4 Division of Endocrinology, Department of Medicine, NYC Health + Hospitals/Harlem, New York, NY 10037, USA; 5 Division of Gastroenterology, Department of Medicine, NYC Health + Hospitals/Harlem, New York, NY 10037, USA

**Keywords:** food insecurity, neighborhood deprivation, social determinants of health, MASLD, NAFLD, health inequities, health policy

## Abstract

**Background/Objectives:** Social determinants of health are implicated in metabolic dysfunction–associated steatotic liver disease (MASLD), yet longitudinal evidence on incident disease is scarce, and whether individual- and community-level determinants act independently remains unclear. The objective of this study was to examine whether individual food insecurity and neighborhood deprivation are independently associated with incident MASLD. **Methods:** In this retrospective cohort of 40,693 U.S. adults without baseline MASLD in the diverse NIH *All of Us* Research Program, food insecurity (two-item Hunger Vital Sign) and neighborhood deprivation (validated five-domain composite) were co-primary exposures, with incident MASLD ascertained from validated diagnostic codes. Proportional hazards models were adjusted for demographic, socioeconomic, and metabolic factors, including obesity. **Results:** Over 116,220 person-years, 1240 (3.0%) participants developed MASLD. Both associations remained significant after adjustment for demographic, socioeconomic, and major metabolic risk factors, including obesity (food insecurity adjusted hazard ratio [aHR] 1.32, 95% CI 1.13–1.53; neighborhood deprivation aHR 1.20, 95% CI 1.05–1.37). Unadjusted cumulative incidence was highest among participants with both exposures and lowest among those with neither. Findings were consistent across sensitivity analyses; the food-insecurity association remained when neighborhood disadvantage was specified with a census-based index. **Conclusions:** Food insecurity and neighborhood deprivation were independently associated with higher incidence of MASLD. Nutrition-focused, equity-oriented interventions warrant evaluation to reduce MASLD burden and advance health equity.

## 1. Introduction

Metabolic dysfunction-associated steatotic liver disease (MASLD), the term adopted in 2023 to replace nonalcoholic fatty liver disease (NAFLD), has become the most common chronic liver disease worldwide, affecting approximately one-third of the adult population [1,2]. Its prevalence has climbed steadily over the past three decades alongside the global rise in obesity and type 2 diabetes, and MASLD now affects up to 65% of individuals living with type 2 diabetes [3,4]. Once regarded as a purely hepatic condition, it is now understood as a systemic disorder associated with complications that extend well beyond the liver, including myocardial infarction and stroke, heart failure, chronic kidney disease, atrial fibrillation, hepatocellular carcinoma, and extrahepatic malignancies [5,6,7,8,9,10,11,12]. Disease risk is multifactorial: PNPLA3 genetic variants are associated with increased hepatic fat and MASLD risk, especially in Hispanic populations, and higher risk-allele frequency among some Mexican-American communities relative to many Caribbean Hispanic groups has been linked to greater Native American ancestry [13,14,15]. In addition, sedentary lifestyle and other behavioral factors, metabolic alterations, environmental exposures, and socioeconomic conditions interact to shape susceptibility and clinical presentation [6,16]. Because approved therapies remain limited and the disease often progresses silently, curbing this multisystem burden depends on identifying and modifying the upstream factors that contribute to its onset [6]. Like other cardiometabolic conditions, MASLD is unevenly distributed and is increasingly understood to be influenced by social and structural inequities [16]. This has shifted attention toward the social determinants of health.

Food insecurity, defined as limited or uncertain access to adequate, safe, and nutritious food, is among the most pervasive and modifiable individual-level social determinants of health, affecting an estimated 2.4 billion people globally and roughly 13.5% of U.S. households [17]. In the United States, food insecurity falls disproportionately on low-income households and on racial and ethnic minority populations—including non-Hispanic Black and Hispanic communities—that experience higher rates of economic hardship and structural barriers to nutritious food access [18]. Several biologically plausible pathways link food insecurity to metabolic and liver disease: constrained food budgets favor inexpensive, energy-dense, nutrient-poor, and ultra-processed foods that promote insulin resistance, hepatic lipid accumulation, and systemic inflammation [19], while the chronic stress of scarcity can dysregulate hypothalamic–pituitary–adrenal axis activity and reinforce maladaptive eating and sedentary behaviors [20,21]. Conversely, higher diet quality and greater physical activity are associated with lower MASLD risk [22]. Accordingly, cross-sectional studies have linked food insecurity to a higher prevalence of MASLD, more advanced hepatic fibrosis, and greater liver-related mortality [17,18,23,24]. Because these studies assessed exposure and disease at a single point in time, they cannot establish whether food insecurity precedes, and may therefore contribute to, the onset of new disease.

Beyond the individual, the community context exerts its own influence. While food insecurity operates largely at the household level, neighborhood deprivation captures the community-level environment in which metabolic risk accumulates—a disadvantaged physical and social setting marked by limited walkability, perceived crime and safety concerns, physical and social disorder, and low social cohesion, set against a backdrop of socioeconomic disadvantage. Such environments concentrate structural barriers to metabolic health: limited access to affordable, nutritious food, under-resourced or unsafe surroundings that discourage physical activity, and chronic environmental stressors [25,26,27]. Many of these patterns trace to historical redlining and disinvestment that entrenched disadvantages in predominantly Black and Hispanic neighborhoods and still shape present-day food environments [28]. Consistent with this, neighborhood deprivation has been associated with greater MASLD severity, steatohepatitis, cirrhosis, healthcare utilization, and mortality [16,25,26,29,30]. Yet, food insecurity and neighborhood deprivation frequently co-occur within the same disadvantaged populations, and prior work has rarely modeled them together, leaving unclear whether community-level deprivation is associated with MASLD independently of individual-level food insecurity and of established metabolic risk factors such as obesity.

Addressing these gaps is essential for identifying modifiable, upstream targets for prevention and for advancing equity in liver health. Using the National Institutes of Health *All of Us* Research Program, a large, diverse, nationally drawn U.S. cohort with linked survey and electronic health record data [31], we conducted a retrospective cohort study to determine whether food insecurity and neighborhood deprivation are each independently associated with incident MASLD among U.S. adults free of the disease at baseline, and whether their co-occurrence is associated with graded risk beyond either exposure alone, after accounting for demographic, socioeconomic, and metabolic factors.

## 2. Materials and Methods

### 2.1. Data Source and Study Population

**Figure 1:**
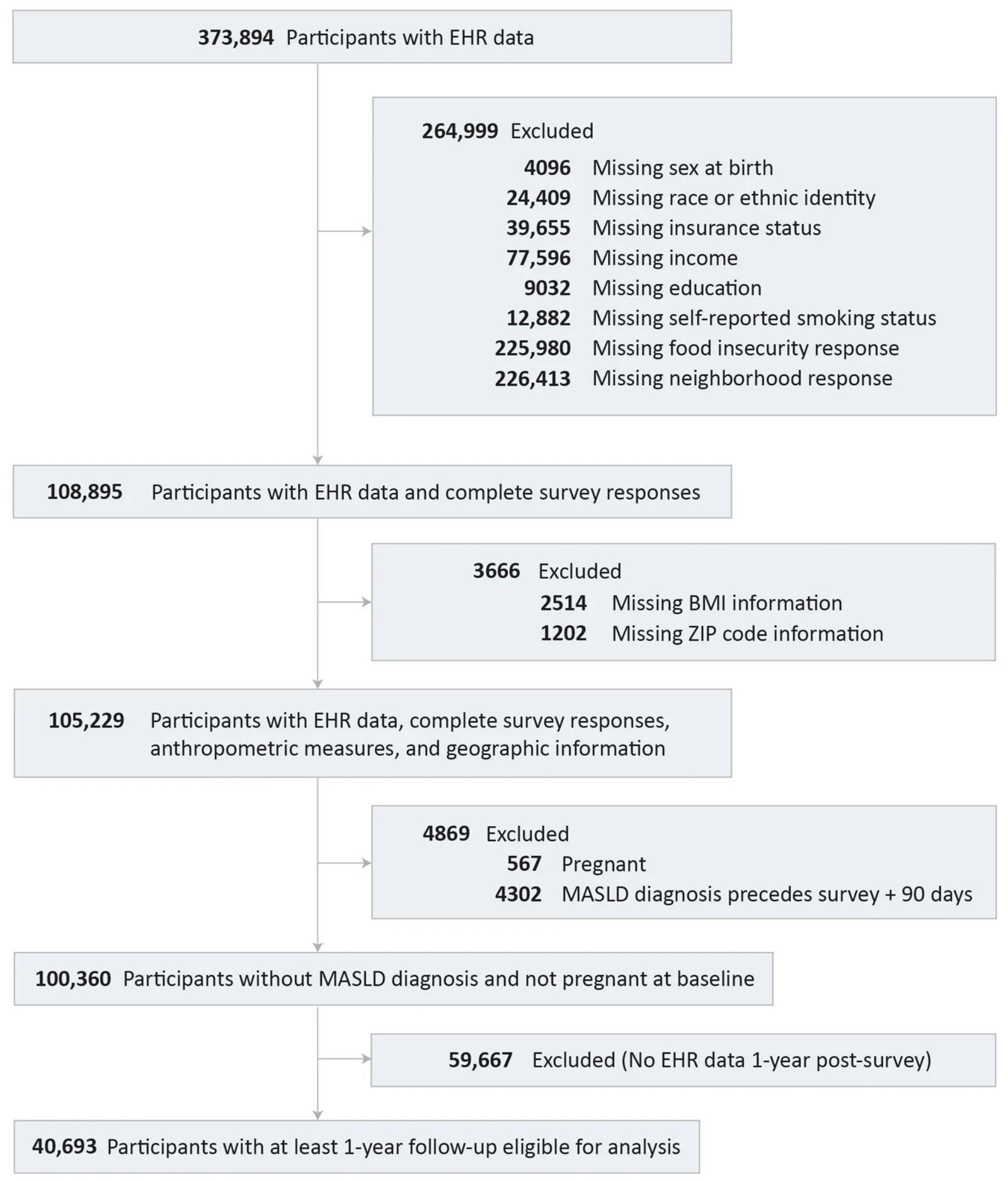
Flowchart of study population selection. BMI: body mass index; EHR: electronic health record; MASLD: metabolic dysfunction-associated steatotic liver disease.

This retrospective cohort study used the Controlled Tier Dataset Version 8 (CDR v8) from the *All of Us* Research Program, a large-scale cohort initiated by the NIH. This extract included longitudinal electronic health records (EHRs) for 373,894 adults living in the U.S. (Figure 1). Adults were included if they: (1) completed “The Basics” and “Social Determinants of Health” surveys between May 2017 and October 2023; (2) had linked EHR data; and (3) had complete data on demographics (age, sex, race/ethnicity), socioeconomic factors (insurance category, education, income), smoking status, food insecurity, neighborhood deprivation, body mass index (BMI), and residential three-digit ZIP codes. We excluded individuals with: (1) MASLD diagnosis documented prior to or within 90 days following survey completion; (2) less than one year of EHR follow-up after the survey; (3) missing data for study variables; or (4) pregnancy at baseline (see Figure 1 for the study flowchart). Participants who selected “prefer not to answer” for race/ethnicity were also excluded because the small numbers in these subgroups prevented reliable statistical analysis.

The *All of Us* Research Program Institutional Review Board approved the study protocol [31]. All participants provided informed consent. Because the data used were de-identified, the study did not require additional institutional review board review.

### 2.2. Exposure and Covariate Assessment

The study had two primary exposure variables: food insecurity and neighborhood deprivation. Food insecurity was determined using the validated two-item Hunger Vital Sign questionnaire from the United States Adult Food Security Survey Module [32,33]. These questions are highly sensitive (≥97%) and reasonably specific (≥74%) for detecting food insecurity [34]. Neighborhood deprivation was assessed using a composite measure derived from five validated survey instruments: (1) Physical Activity Neighborhood Environment Scale (PANES) walking/bicycling items, (2) PANES crime/safety items, (3) Social Cohesion and Trust Scale, (4) Neighborhood Physical Disorder scale, and (5) Neighborhood Social Disorder scale [35,36,37]. For each domain, participants scoring at or above the 75th percentile were classified as high risk. Participants with ≥3 risk domains were classified as experiencing high neighborhood deprivation. Detailed methodology defining neighborhood deprivation is provided in Supplementary Table S1.

Age was calculated based on the date the participant completed the survey. This date marked their entry into the study cohort and the time at which food insecurity status was assessed. Sex assigned at birth was recorded as male or female. Self-reported race/ethnicity was categorized as Hispanic, non-Hispanic White, non-Hispanic Black, or Other. The “Other” category combined non-Hispanic Asian, Middle Eastern/North African, Native Hawaiian/Other Pacific Islander, American Indian/Alaska Native, and multiracial individuals. Smoking status was self-reported at the time of the survey. Alcohol consumption was assessed using the Alcohol Use Disorders Identification Test-Concise (AUDIT-C) [38]. Individual-level socioeconomic data, including insurance status, income, and education level, were collected from baseline surveys. Insurance status was categorized as no insurance/public insurance versus private insurance. Body mass index (BMI, in kg/m^2^) was categorized based on World Health Organization (WHO) guidelines. For non-Asian participants, overweight was defined as a BMI of 25.0–29.9, and obesity as a BMI ≥ 30.0. For Asian participants, overweight was defined as a BMI of 23.0–24.9, and obesity as a BMI ≥ 25.0 [39,40]. Comorbidities such as hypertension, hyperlipidemia, and type 2 diabetes were identified using a combination of International Classification of Diseases, Ninth and Tenth Revision (ICD-9/10) codes, laboratory results, physical measurements, and medication records from the EHR. Specific criteria and codes are detailed in Supplementary Table S2.

### 2.3. Outcomes

The primary outcome was new-onset, incident MASLD. MASLD was identified by ICD-9/10 codes in the EHR (Supplementary Table S2). To improve specificity, participants were excluded if they had steatosis due to other known causes (e.g., viral hepatitis) or significant alcohol intake. Significant alcohol intake was defined as >140 g/week for females or >210 g/week for males (based on AUDIT-C survey responses), or any alcohol-related disorder ICD codes. This method for identifying MASLD has shown a positive predictive value of 90–91% in prior studies [41,42]. The date of incident MASLD was defined as the date of the first MASLD-related EHR code recorded after the baseline survey.

### 2.4. Statistical Analysis

Baseline characteristics were compared using *t*-tests or Wilcoxon rank-sum tests for continuous data, and Chi-squared or Fisher’s exact tests for categorical data.

The number of incident events (*n* = 1240) was adequate to support the planned multivariable Cox proportional hazards models given the number of covariates.

The follow-up period for each participant began on the date of survey completion and extended until the first occurrence of MASLD diagnosis, the last available EHR record, or the end of data availability in CDR v8 (1 October 2023), whichever occurred first. Participants who did not develop MASLD by the end of this period were censored at that time. The distribution of follow-up time (in days) was non-normal and is therefore reported using medians and interquartile ranges (IQRs). Comparisons of follow-up duration between groups were made using the Wilcoxon rank-sum test with continuity correction.

Cox proportional-hazards models were used to evaluate the association of food insecurity and neighborhood deprivation, each modeled as a binary exposure, with incident MASLD. Models were adjusted for demographic factors (age at enrollment, sex at birth, race/ethnicity), socioeconomic variables (insurance category, education, income), smoking status, and metabolic risk factors (obesity, hypertension, hyperlipidemia, and type 2 diabetes). There was no evidence of a proportional-hazards violation based on Schoenfeld residuals (*p* = 0.77). Statistical significance was assessed at a two-sided *p* < 0.05.

All analyses were conducted in R version 4.4.0 (R Foundation for Statistical Computing, Vienna, Austria) within JupyterLab version 4.5.6 on the *All of Us* Researcher Workbench. To protect privacy and in accordance with the *All of Us* Data and Statistics Dissemination Policy, participant counts ≤ 20 are not presented.

### 2.5. Sensitivity Analyses

We performed sensitivity analyses to verify robustness. To address possible reverse causation, we conducted time-lagged analyses excluding events within the first 6 and 12 months. We enhanced outcome specificity by requiring 2 MASLD diagnostic codes separated by 30 days. To mitigate residual confounding, we employed 1:1 propensity-score matching (caliper 0.20 SD) with doubly robust estimation. Models were also adjusted for birth year to capture secular effects. These analyses were applied to both exposures (Supplementary Tables S4 and S5). In food-insecurity models, we additionally tested a stricter exposure definition (affirmative responses to both questions) and substituted the census-derived Nationwide Community Deprivation Index for the survey-based neighborhood-deprivation covariate (Supplementary Tables S1 and S4) [43].

## 3. Results

### 3.1. Participant Characteristics

Of the 40,693 participants included in the study (Figure 1), 5494 (13.5%) were classified as food-insecure, and 7633 (18.8%) lived in neighborhood-deprived areas. Baseline characteristics stratified by food security status and neighborhood deprivation are presented in Table 1 and Table 2, respectively. Compared with their respective reference groups (food-secure and non–neighborhood-deprived), both exposed groups were generally younger, more often female, and more likely to identify as non-Hispanic Black or Hispanic. They also had higher rates of public or no insurance, lower educational attainment, and lower income. Clinically, both groups reported higher smoking rates and greater prevalence of obesity and type 2 diabetes. Whereas food-insecure individuals had similar rates of hypertension compared with food-secure individuals, neighborhood-deprived individuals had a slightly lower prevalence of hypertension and hyperlipidemia. Baseline characteristics stratified by food security status and neighborhood deprivation are described in Table 1 and Table 2, respectively.

There was considerable overlap between the two exposures: 38.6% of food-insecure participants lived in neighborhood-deprived areas, and 27.8% of neighborhood-deprived participants were food-insecure, underscoring the importance of modeling both determinants jointly.

### 3.2. Incidence of MASLD by Food Insecurity and Neighborhood Deprivation

During the study period (May 2018–October 2023), 1240 (3.0%) incident MASLD cases were identified.

**Figure 2:**
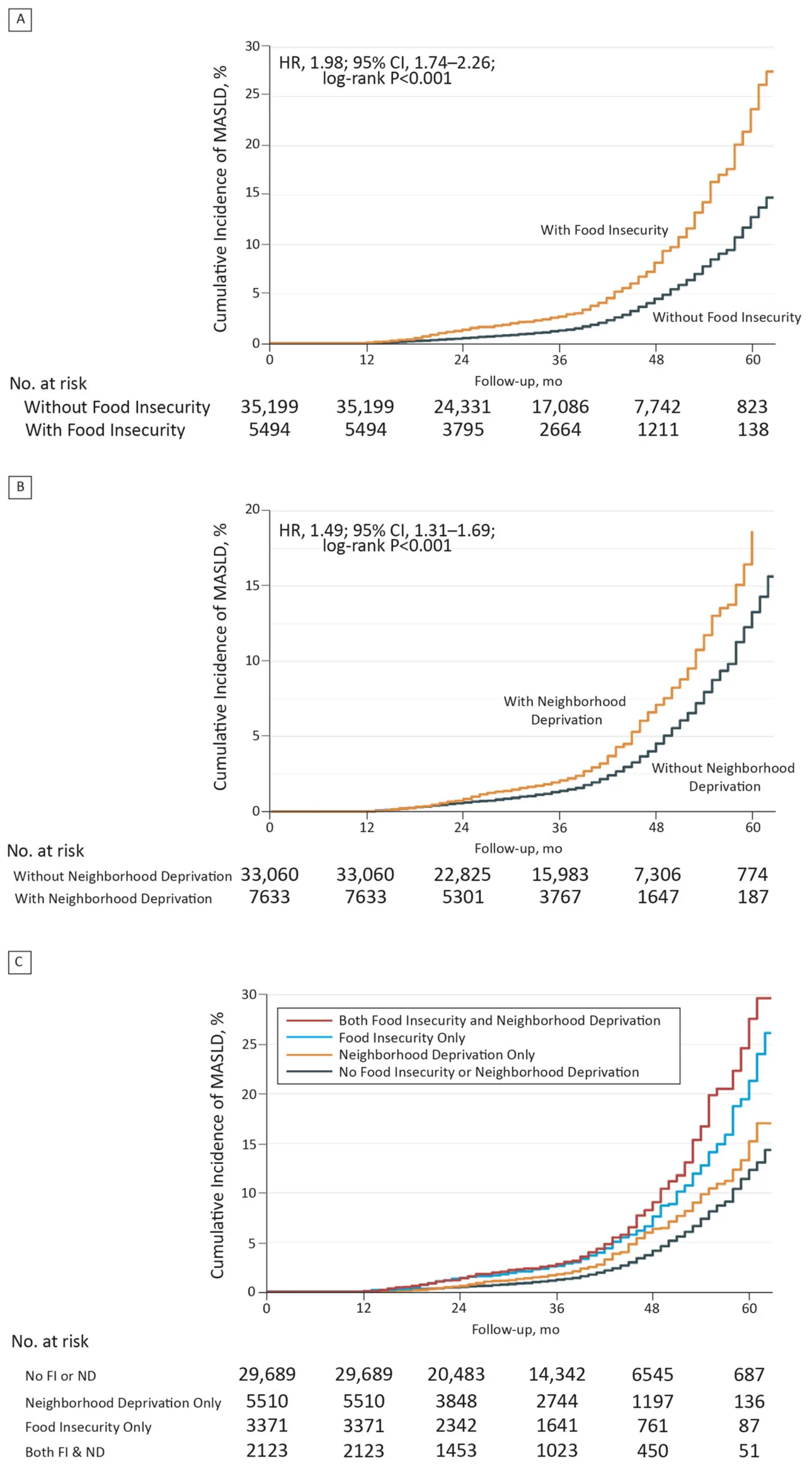
Cumulative incidence of MASLD by food insecurity and neighborhood deprivation status. (**A**) Incident MASLD by food insecurity; (**B**) incident MASLD by neighborhood deprivation; (**C**) incident MASLD by joint food insecurity and neighborhood deprivation status (both exposures; food insecurity only; neighborhood deprivation only; neither). FI: food insecurity; ND: neighborhood deprivation; MASLD: metabolic dysfunction-associated steatotic liver disease; HR: hazard ratio; CI: confidence interval. The hazard ratios depicted in this figure are unadjusted hazard ratios.

In the food-insecure group (*n* = 5494; 15,694 person-years), 292 participants (5.3%) developed MASLD compared with 948 participants (2.7%) in the food-secure group (*n* = 35,199; 100,526 person-years). The incidence rates were 18.6 and 9.43 per 1000 person-years, respectively, yielding an unadjusted HR of 1.98 (95% CI 1.74–2.26) (Figure 2A). After adjustment for demographic, socioeconomic, and metabolic factors—including obesity—the association remained significant (aHR 1.32, 95% CI 1.13–1.53). The median follow-up time was 35 months (IQR, 21–46) in both groups (*p* = 0.91). Among participants who developed MASLD, the median time to diagnosis was 43 months (IQR, 31–50) in the food-secure group and 42 months (IQR, 28–49) in the food-insecure group (*p* = 0.34).

In the neighborhood-deprived group (*n* = 7633; 21,875 person-years), 319 participants (4.2%) developed MASLD compared with 921 participants (2.8%) in the non–neighborhood-deprived group (*n* = 33,060; 94,345 person-years). The corresponding incidence rates were 14.6 and 9.76 per 1000 person-years, with an unadjusted HR of 1.49 (95% CI 1.31–1.69) (Figure 2B). After full adjustment, neighborhood deprivation remained independently associated with incident MASLD (aHR 1.20, 95% CI 1.05–1.37). The median follow-up time was 35 months (IQR, 21–46) in both groups (*p* = 0.45). Among participants who developed MASLD, the median time to diagnosis was 43 months (IQR, 31–50) in the non–neighborhood-deprived group and 42 months (IQR, 30–49) in the neighborhood-deprived group (*p* = 0.55).

When the two exposures were considered jointly, cumulative MASLD incidence rose in a graded fashion across mutually exclusive groups (Figure 2C): incidence was lowest among participants with neither exposure, higher among those with neighborhood deprivation alone, higher still among those with food insecurity alone, and highest among those experiencing both food insecurity and neighborhood deprivation. This unadjusted pattern is consistent with a graded contribution of the two social determinants to subsequent MASLD diagnosis.

### 3.3. Factors Associated with MASLD Incidence

**Figure 3:**
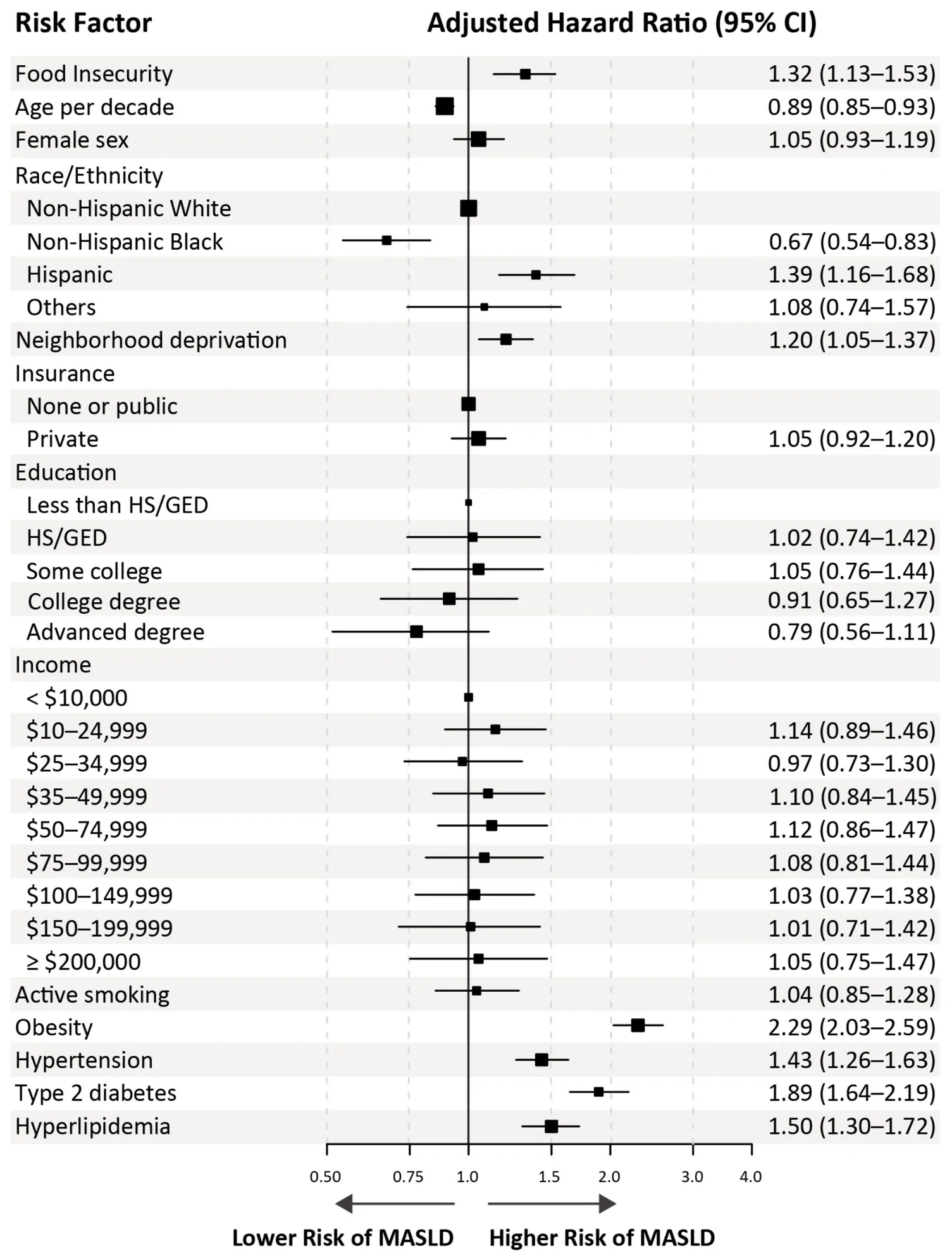
Forest plot of adjusted associations with incident MASLD. MASLD, metabolic dysfunction-associated steatotic liver disease; HS/GED, high school/General Educational Development; CI, confidence interval; HR, hazard ratio.

In the adjusted model, food insecurity and neighborhood deprivation remained independently associated with incident MASLD. In addition, Hispanic ethnicity, obesity, hypertension, hyperlipidemia, and type 2 diabetes were independently associated with increased risk of MASLD; obesity showed the strongest association in the multivariable model. By contrast, age and non-Hispanic Black race/ethnicity were inversely associated with MASLD. Full univariable and multivariable results are presented in Supplementary Table S3 and Figure 3, which summarize the relative magnitude of social and metabolic associations.

### 3.4. Sensitivity Analyses

Associations remained generally consistent across sensitivity analyses, including time-lagged and propensity-score–matched models (Supplementary Tables S4 and S5); the neighborhood association was attenuated and no longer statistically significant under the two-code MASLD definition. The association with food insecurity persisted using stricter definitions for both MASLD (aHR 1.44, 95% CI 1.15–1.80) and food insecurity (aHR 1.25, 95% CI 1.04–1.49). The food-insecurity association also persisted when neighborhood disadvantage was instead specified with the census-derived Nationwide Community Deprivation Index (aHR 1.34, 95% CI 1.15–1.56).

## 4. Discussion

In this large retrospective cohort of U.S. adults free of MASLD at baseline, food insecurity and neighborhood deprivation were each independently associated with higher incidence of subsequent MASLD diagnosis after adjustment for demographic, socioeconomic, and metabolic factors, including obesity. When modeled jointly, each exposure retained an independent association, and unadjusted cumulative incidence rose in a graded fashion from neither exposure to both. Estimates were stable across multiple sensitivity analyses, including lagged outcomes, a stricter outcome definition, propensity-score matching, and, for food insecurity, a stricter exposure definition and substitution of a census-based deprivation covariate. These findings support a temporal association between two complementary social determinants and incident MASLD, while not establishing causation.

Our study advances a largely cross-sectional literature in several ways. By focusing on incident rather than prevalent disease in a cohort free of MASLD at entry, it supports temporality that prior work linking food insecurity or community deprivation to MASLD prevalence, fibrosis, and mortality could not [16,17,18,23,24,25,26,29,30]. To our knowledge, it is also among the first analyses to model an individual-level determinant (food insecurity) and a community-level determinant (neighborhood deprivation) within one framework, showing that each is associated with incident MASLD independently of the other and of metabolic factors such as obesity and type 2 diabetes. The food-insecurity association was also unchanged when the survey-based neighborhood covariate was replaced with an administrative census index, indicating that this finding was not dependent on a single measure of community disadvantage [43]. The joint-exposure pattern further suggests that co-occurring household and community disadvantage may compound risk for subsequent diagnosis.

Several mechanisms plausibly link food insecurity to hepatic steatosis. Constrained food budgets shift diets toward inexpensive, energy-dense, nutrient-poor foods, including ultra-processed products, that promote insulin resistance, hepatic lipid accumulation, and systemic inflammation—the core pathogenic mechanisms of MASLD. Dietary patterns most protective against steatotic liver disease then become harder to sustain [19,22]. Dietary quality and ultra-processed food consumption are therefore leading candidate mediators; these variables were not directly available in the present dataset and should be prioritized in future mediation analyses. Superimposed on these nutritional pathways, the chronic stress of scarcity can dysregulate hypothalamic–pituitary–adrenal axis activity and favor visceral and hepatic fat deposition while entrenching maladaptive eating and inactivity [20,21]. That food insecurity remained independently associated with incident MASLD after adjustment for obesity, type 2 diabetes, and other metabolic factors suggests that these pathways may act partly upstream of, or in parallel with, conventional cardiometabolic risk factors.

The independent association of neighborhood deprivation points to structural drivers beyond the household. Deprived communities are disproportionately food deserts and food swamps—environments recently linked to higher MASLD mortality at the U.S. county level [27]. They offer fewer safe, walkable spaces for activity and greater exposure to stressors, reinforcing the pathways above [20,21,25,26]. These disadvantages are partly the legacy of discriminatory practices such as redlining, which channeled disinvestment away from predominantly Black and Hispanic neighborhoods and entrenched the food environments that persist today [28]. Because such forces concentrate food insecurity and neighborhood deprivation together, risk may accumulate where they converge. That food insecurity alone was associated with a higher unadjusted incidence than neighborhood deprivation alone likely reflects the more proximal influence of household food access on daily diet.

Perceived and objective neighborhood measures capture related but non-identical constructs. Our primary deprivation metric was derived from validated survey instruments reflecting participants’ lived experience of walkability, safety, disorder, and social cohesion—features that may more directly influence daily physical activity, stress, and food acquisition. In contrast, the census-derived Nationwide Community Deprivation Index summarizes area-level socioeconomic conditions (poverty, income, education, insurance, public assistance, and housing vacancy) and is less susceptible to same-source reporting bias, but it may misclassify individual experience through ecological aggregation and imperfect linkage from three-digit ZIP codes to census tracts [43]. Neither approach fully captures the multidimensional neighborhood environment. Because the census index was used as an alternative community-level covariate in the food-insecurity models rather than as a replacement primary exposure, these complementary measures inform interpretation of the food-insecurity association but do not constitute an independent replication of the neighborhood-deprivation finding.

Our model was also consistent with the established demographic epidemiology of MASLD. Hispanic ethnicity was independently associated with elevated risk, consistent with evidence that Native American ancestry and the PNPLA3 variant heighten susceptibility, particularly in some Hispanic subgroups [13,14,15]. Obesity remained the strongest associated factor, with more than 2-fold increased risk, followed by type 2 diabetes, hyperlipidemia, and hypertension, underscoring that social determinants act alongside—not instead of—metabolic drivers. Two inverse associations warrant caution rather than a reading of protection. The lower adjusted risk among non-Hispanic Black participants aligns with the lower burden of hepatic steatosis reported in populations of African ancestry [13,15]. Still, it may partly reflect differential care access and EHR ascertainment, especially as this group carried a disproportionate baseline burden of both exposures. The inverse association with age most plausibly reflects survivorship, competing mortality, the baseline exclusion of prevalent cases, and possibly secular changes in diet over time, rather than a protective effect of aging, and underscores that young-onset disease is often more aggressive [44].

Interpretation should remain cautious. Food insecurity and neighborhood deprivation are multidimensional constructs that may interact with numerous biological, behavioral, environmental, and healthcare-related factors. Despite extensive adjustment and propensity-score matching, residual and unmeasured confounding cannot be excluded. Unmeasured or incompletely measured factors may include healthcare access and engagement, dietary quality and ultra-processed food intake, employment and working conditions, chronic stress, physical activity, detailed built-environment features, transportation, medication use, genetic predisposition beyond population ancestry proxies, and other structural determinants of health. Accordingly, our estimates should be read as adjusted associations with subsequent MASLD diagnosis, not as causal effects. These findings nonetheless have clinical, nutritional, and policy implications that require prospective validation. Both exposures were common in this cohort—affecting 13.5% and 18.8% of participants, respectively—so even moderate per-person associations could translate into a meaningful population-level burden of MASLD. Because food insecurity is readily identified with the two-item Hunger Vital Sign, incorporating it into routine screening of patients at metabolic risk could flag a modifiable vulnerability and prompt referral to dietitians, food-assistance resources, and stress-management support [17,18,20,21,23,24].

The emerging “Food is Medicine” paradigm—medically tailored meals, produce prescriptions, and clinic-linked food pharmacies—is mechanistically aligned with the diet-quality pathway [22]. At the population level, strengthening the reach and nutritional quality of programs such as the Supplemental Nutrition Assistance Program (SNAP), alongside measures that curb food swamps and expand access to affordable, nutritious food and safe spaces for activity, targets structural drivers of neighborhood risk [16,25,27,28,29,30]. Because participants with both exposures had the highest unadjusted cumulative incidence, strategies that address household food access and the surrounding food environment together merit evaluation. We emphasize, however, that none of these interventions has yet been shown to prevent MASLD; residual confounding remains possible, and our observational data motivate—but cannot replace—interventional trials.

Key strengths include the longitudinal, incident-outcome design with exposures ascertained before disease onset; a large, diverse, nationally recruited cohort that enhances generalizability; linkage of validated survey measures of social determinants to longitudinal EHR data, permitting mutual adjustment of two conceptually distinct determinants; a coding algorithm with a positive predictive value of 90–91% [41,42]; and consistency of the food-insecurity association across lagged, matched, stricter-definition, and alternative-covariate models, together with a doubly robust matched analysis of neighborhood deprivation. Several limitations temper interpretation. MASLD was identified from ICD-9/10 codes, which, despite their high positive predictive value, remain susceptible to misclassification and underdiagnosis—particularly among individuals with limited healthcare engagement, precisely the disadvantaged groups of interest—so the observed racial and ethnic differences may partly reflect differential access to care; a stricter two-code definition yielded consistent estimates for food insecurity, whereas the neighborhood association was attenuated and no longer statistically significant. Despite extensive adjustment and propensity-score matching, residual confounding from unmeasured factors cannot be excluded, as detailed above. The two-item food-insecurity screen does not grade severity or chronicity, and both food insecurity and neighborhood deprivation were assessed only once at baseline; exposures may have changed during follow-up, potentially leading to non-differential exposure misclassification and bias toward the null. Finally, requiring complete survey and EHR data excluded some participants and may have underrepresented the most vulnerable individuals; if anything, this would bias estimates toward the null. As an observational study, our analysis supports a temporal association, and further studies are needed to establish causation.

## 5. Conclusions

In this diverse U.S. cohort, food insecurity and neighborhood deprivation were independently associated with higher incidence of subsequent MASLD diagnosis, with the highest unadjusted cumulative incidence among adults experiencing both forms of disadvantage. These findings identify modifiable nutritional and structural social determinants that warrant attention in clinical screening, community nutrition programs, and health-equity policy. Multilevel strategies that pair individual screening and nutritional support with community and policy action merit rigorous prospective and interventional evaluation to reduce the burden of MASLD and advance health equity.

## Figures and Tables

**Table 1:** Baseline characteristics stratified by food security status.

Characteristics	Overall, *n* = 40,693	Food-Secure, *n* = 35,199	Food-Insecure, *n* = 5494	*p* Value
Age in years, mean (SD)	57.6 (15.5)	58.7 (15.3)	50.6 (14.9)	<0.001
Male sex	14,430 (35.5)	12,984 (36.9)	1446 (26.3)	<0.001
Race/ethnicity				<0.001
Non-Hispanic White	33,254 (81.7)	29,878 (84.9)	3376 (61.4)	
Non-Hispanic Black	3267 (8.0)	2164 (6.1)	1103 (20.1)	
Hispanic	3103 (7.6)	2187 (6.2)	916 (16.7)	
Other (Non-Hispanic Asian, MENA, NHPI, AIAN)	1069 (2.6)	970 (2.8)	99 (1.8)	
Insurance: None or public	18,653 (45.8)	15,115 (42.9)	3538 (64.4)	<0.001
Education				<0.001
Less than HS/GED	836 (2.1)	472 (1.3)	364 (6.6)	
HS/GED	3807 (9.4)	2694 (7.7)	1113 (20.3)	
Some college	9791 (24.1)	7575 (21.5)	2216 (40.3)	
College degree	12,075 (29.7)	10,942 (31.1)	1133 (20.6)	
Advanced degree	14,184 (34.9)	13,516 (38.4)	668 (12.2)	
Annual income				<0.001
<$10,000	2242 (5.5)	1107 (3.1)	1135 (20.7)	
$10–24,999	3835 (9.4)	2293 (6.5)	1542 (28.1)	
$25–34,999	2901 (7.1)	2164 (6.1)	737 (13.4)	
$35–49,999	4038 (9.9)	3314 (9.4)	724 (13.2)	
$50–74,999	6628 (16.3)	5945 (16.9)	683 (12.4)	
$75–99,999	5578 (13.7)	5248 (14.9)	330 (6.0)	
$100–149,999	7308 (18.0)	7085 (20.1)	223 (4.1)	
$150–199,999	3425 (8.4)	3362 (9.6)	63 (1.1)	
≥$200,000	4738 (11.6)	4681 (13.3)	57 (1.0)	
Neighborhood deprivation	7633 (18.8)	5510 (15.7)	2123 (38.6)	<0.001
Current smoker	2846 (7.0)	1763 (5.0)	1083 (19.7)	<0.001
BMI, mean (SD)	29.2 (7.0)	28.7 (6.6)	32.3 (8.6)	<0.001
Weight category				<0.001
Healthy	11,535 (28.3)	10,527 (29.9)	1008 (18.3)	
Underweight	511 (1.3)	445 (1.3)	66 (1.2)	
Overweight	13,168 (32.4)	11,805 (33.5)	1363 (24.8)	
Obesity, class 1	8367 (20.6)	7075 (20.1)	1292 (23.5)	
Obesity, class 2	4085 (10.0)	3220 (9.1)	865 (15.7)	
Obesity, class 3	3027 (7.4)	2127 (6.0)	900 (16.4)	
Obesity	15,479 (38.0)	12,422 (35.3)	3057 (55.6)	<0.001
Hypertension	13,696 (33.7)	11,824 (33.6)	1872 (34.1)	0.492
Type 2 diabetes	3580 (8.8)	2849 (8.1)	731 (13.3)	<0.001
Hyperlipidemia	14,433 (35.5)	12,652 (35.9)	1781 (32.4)	<0.001

HS/GED: high school/General Educational Development; MENA: Middle Eastern and North African; NHPI: Native Hawaiian and Other Pacific Islander; AIAN: American Indian and Alaska Native; SD: standard deviation; BMI: body mass index. Values are *n* (%) unless otherwise indicated.

**Table 2:** Baseline characteristics stratified by neighborhood deprivation.

Characteristics	Overall, *n* = 40,693	Non-Neighborhood-Deprived, *n* = 33,060	Neighborhood-Deprived, *n* = 7633	*p* Value
Age in years, mean (SD)	57.6 (15.5)	58.6 (15.3)	53.7 (15.7)	<0.001
Male sex	14,430 (35.5)	11,855 (35.9)	2575 (33.7)	<0.001
Race/ethnicity				<0.001
Non-Hispanic White	33,254 (81.7)	27,865 (84.3)	5389 (70.6)	
Non-Hispanic Black	3267 (8.0)	2141 (6.5)	1126 (14.8)	
Hispanic	3103 (7.6)	2155 (6.5)	948 (12.4)	
Other (Non-Hispanic Asian, MENA, NHPI, AIAN)	1069 (2.6)	899 (2.7)	170 (2.2)	
Insurance: None or public	18,653 (45.8)	14,657 (44.3)	3996 (52.4)	<0.001
Education				<0.001
Less than HS/GED	836 (2.1)	470 (1.4)	366 (4.8)	
HS/GED	3807 (9.4)	2608 (7.9)	1199 (15.7)	
Some college	9791 (24.1)	7419 (22.4)	2372 (31.1)	
College degree	12,075 (29.7)	10,119 (30.6)	1956 (25.6)	
Advanced degree	14,184 (34.9)	12,444 (37.6)	1740 (22.8)	
Annual income				<0.001
<$10,000	2242 (5.5)	1292 (3.9)	950 (12.4)	
$10–24,999	3835 (9.4)	2556 (7.7)	1279 (16.8)	
$25–34,999	2901 (7.1)	2128 (6.4)	773 (10.1)	
$35–49,999	4038 (9.9)	3164 (9.6)	874 (11.5)	
$50–74,999	6628 (16.3)	5406 (16.4)	1222 (16.0)	
$75–99,999	5578 (13.7)	4719 (14.3)	859 (11.3)	
$100–149,999	7308 (18.0)	6421 (19.4)	887 (11.6)	
$150–199,999	3425 (8.4)	3062 (9.3)	363 (4.8)	
≥$200,000	4738 (11.6)	4312 (13.0)	426 (5.6)	
Food insecurity	5494 (13.5)	3371 (10.2)	2123 (27.8)	<0.001
Current smoker	2846 (7.0)	1866 (5.6)	980 (12.8)	<0.001
BMI, mean (SD)	29.2 (7.0)	28.8 (6.7)	30.6 (8.0)	<0.001
Weight category				<0.001
Healthy	11,535 (28.3)	9742 (29.5)	1793 (23.5)	
Underweight	511 (1.3)	406 (1.2)	105 (1.4)	
Overweight	13,168 (32.4)	10,945 (33.1)	2223 (29.1)	
Obesity, class 1	8367 (20.6)	6739 (20.4)	1628 (21.3)	
Obesity, class 2	4085 (10.0)	3067 (9.3)	1018 (13.3)	
Obesity, class 3	3027 (7.4)	2161 (6.5)	866 (11.3)	
Obesity	15,479 (38.0)	11,967 (36.2)	3512 (46.0)	<0.001
Hypertension	13,696 (33.7)	11,204 (33.9)	2492 (32.6)	0.040
Type 2 diabetes	3580 (8.8)	2772 (8.4)	808 (10.6)	<0.001
Hyperlipidemia	14,433 (35.5)	11,947 (36.1)	2486 (32.6)	<0.001

HS/GED: high school/General Educational Development; MENA: Middle Eastern and North African; NHPI: Native Hawaiian and Other Pacific Islander; AIAN: American Indian and Alaska Native; SD: standard deviation; BMI: body mass index. Values are *n* (%) unless otherwise indicated.

## Data Availability

This study used data from the *All of Us* Research Program, a publicly available resource supported by the National Institutes of Health. Data access requires institutional approval and registration through the Researcher Workbench (https://workbench.researchallofus.org/, accessed on 21 July 2026). Permission to access and analyze these data was obtained in accordance with program policies. Data supporting the findings of this study are available from the corresponding author on reasonable request.

## Supplementary Materials

The following supporting information can be downloaded at https://www.mdpi.com/article/10.3390/nu18162741/s1. Table S1: Neighborhood social determinants of health assessment methodology; Table S2: Criteria for defining cardiometabolic conditions and metabolic dysfunction-associated steatotic liver disease (MASLD); Table S3: Univariable and multivariable hazard ratios for MASLD based on demographic, socioeconomic, and metabolic variables; Table S4: Sensitivity analyses for the association between food insecurity and incident MASLD; Table S5: Sensitivity analyses for the association between neighborhood deprivation and incident MASLD.

## Abbreviations

The following abbreviations are used in this manuscript:

AIAN	American Indian and Alaska Native
aHR	Adjusted hazard ratio
AUDIT-C	Alcohol Use Disorders Identification Test-Concise
BMI	Body mass index
CDR	Controlled Tier Dataset
CI	Confidence interval
EHR	Electronic health record
FI	Food insecurity
HR	Hazard ratio
HS/GED	High School/General Educational Development
ICD-9/10	International Classification of Diseases, Ninth and Tenth Revision
IQR	Interquartile range
MASLD	Metabolic dysfunction-associated steatotic liver disease
MENA	Middle Eastern and North African
NAFLD	Nonalcoholic fatty liver disease
ND	Neighborhood deprivation
NHPI	Native Hawaiian and Other Pacific Islander
NIH	National Institutes of Health
PANES	Physical Activity Neighborhood Environment Scale
PNPLA3	Patatin-like phospholipase domain-containing protein 3
SD	Standard deviation
SNAP	Supplemental Nutrition Assistance Program
WHO	World Health Organization
