# Supplementary material for: Independent Associations of Food Insecurity and Neighborhood Deprivation: Independent Associations with Incident Metabolic Dysfunction–Associated Steatotic Liver Disease in a Diverse U.S. Cohort
Source: Nutrients. 2026 Aug 21;18(16):2741. doi: 10.3390/nu18162741 (PMC13516955; doi:10.3390/nu18162741)
Supplement: Supplementary file 1 [file nutrients-18-02741-s001.zip › nutrients-4483559-supplementary.pdf]

Supplementary Table S1. Neighborhood Social Determinants of Health Assessment Methodology

A. Component Measures of Composite Neighborhood Risk Score

Domain	Instrument	Description	Scoring	Risk Classification
Physical Activity Environment	Physical Activity Neighborhood Environment Scale (PANES) – Walking/Bicycling items	Assessed built environment support for physical activity including sidewalk availability, safety for walking/cycling, and access to recreational facilities	Individual items summed to create total score; higher scores = greater barriers to physical activity	High risk: ≥75th percentile
Neighborhood Safety	PANES – Crime & Safety items	Captured perceptions of neighborhood safety, including concerns about crime and personal security	Items summed to create total score; higher scores = greater safety concerns	High risk: ≥75th percentile
Social Cohesion	Social Cohesion and Trust Scale	Assessed neighborhood social connectedness, mutual trust among neighbors, and collective efficacy	Items summed to create total score; higher scores = lower social cohesion/greater fragmentation	High risk: ≥75th percentile
Neighborhood Physical Disorder	Neighborhood Physical Disorder Scale	Measured visible signs of physical deterioration including litter, vandalism, abandoned buildings, and poor property maintenance	Items summed to create total score; higher scores = greater physical disorder	High risk: ≥75th percentile
Neighborhood Social Disorder	Neighborhood Social Disorder Scale	Assessed social problems including public drug use, public intoxication, harassment, and antisocial behaviors	Items summed to create total score; higher scores = greater social disorder	High risk: ≥75th percentile

B. Composite Risk Score Construction

Variable	Definition	Range	Interpretation
Binary indicators	Each domain scored as 1 (≥75th percentile) or 0 (<75th percentile); missing values coded as 0	0–1 per domain	Conservative approach avoiding exclusion of participants with incomplete data
Composite score	Sum of binary indicators across all five domains	0–5	Higher scores indicate multiple concurrent neighborhood disadvantages
Neighborhood deprivation	High neighborhood risk exposure (primary analysis variable)	Binary	≥3 risk factors present

C. Alternative Community-Disadvantage Covariate: Geographic Neighborhood Deprivation

Measure	Data Source	Components	Scoring Method	Classification
Nationwide Community Deprivation Index	2018 American Community Survey (5-year estimates)	Six census tract-level socioeconomic indicators:<br>• Poverty rate: Fraction of households with income below poverty level within the past 12 months<br>• Median household income: Median household income in the past 12 months (2018 inflation-adjusted dollars)<br>• High school completion rate: Fraction of population 25 and older with educational attainment of at least high school graduation (includes GED equivalency)<br>• Health insurance coverage: Fraction of population with health insurance<br>• Public assistance receipt: Fraction of households receiving public assistance income or food stamps/SNAP in the past 12 months<br>• Housing vacancy rate: Fraction of houses that are vacant	Principal component analysis (PCA) of six indicators; first component captures >52% of variance and serves as deprivation index; score rescaled and normalized to range 0–1 (higher = more deprived)	Deprived neighborhood: ≥2018 national median (upper 50th percentile)
Linkage method	Three-digit ZIP codes	ZIP Code Tabulation Area values calculated as mean of all intersecting census tracts	Alternative community-level covariate in food-insecurity models (Table S4)	Not used as the primary neighborhood-deprivation exposure (Table S5)

Note: All survey-based instruments were administered as part of the All of Us Social Determinants of Health questionnaire. This census-derived index was used as an alternative community-level covariate in food-insecurity models (Supplementary Table S4), not as a replacement primary exposure for neighborhood deprivation (Supplementary Table S5).

Abbreviations: GED: General Educational Development

Supplementary Table S2. Criteria for defining cardiometabolic conditions and metabolic dysfunction-associated steatotic liver disease (MASLD)

Condition	Definition Criteria
**Type 2 diabetes**	**Required:** Both of the following:<br>1. At least one drug exposure for type 2 diabetes medications (metformin, sulfonylureas, thiazolidinediones, DPP-4 inhibitors, SGLT-2 inhibitors, GLP-1 receptor agonists).<br>2. At least one qualifying laboratory result:<br>• Random glucose > 200 mg/dL<br>• Fasting glucose ≥ 125 mg/dL<br>• glycated hemoglobin (HbA1c) ≥ 6.5%<br>**Exclusion:** Diagnosis codes for Type 1 diabetes.
**Hypertension**	**Required:** Both of the following:<br>1. At least one diagnosis code for hypertension:<br>• ICD-9: 401, 401.0, 401.1, 401.9<br>• ICD-10: I10<br>2. At least one qualifying blood pressure measurement:<br>• Systolic BP ≥ 130 mmHg<br>• Diastolic BP ≥ 80 mmHg
**Hyperlipidemia**	**Required:** Both of the following:<br>1. At least one diagnosis code for hyperlipidemia:<br>• ICD-9: 272.0, 272.1, 272.2, 272.3, 272.4, 272.9<br>• ICD-10: E78.0, E78.00, E78.01, E78.1, E78.2, E78.3, E78.4, E78.41, E78.49, E78.5, E78.9

	2. At least one drug exposure for lipid-lowering medications (statins: atorvastatin, fluvastatin, lovastatin, pravastatin, rosuvastatin, simvastatin).
**Metabolic Dysfunction-Associated Steatotic Liver Disease (Inclusion Criteria)**	**Required:** At least one of the following diagnosis codes:<br>• ICD-9: 571.8<br>• ICD-10: K76.0<br>**AND** no evidence of excessive alcohol consumption:<br>Defined as:<br>• >140 g/week for females<br>• >210 g/week for males<br>**OR**<br>• Consuming ≥6 drinks daily
**Exclusion Criteria for MASLD (Alternative Etiologies)**	**Any of the following conditions exclude the diagnosis of MASLD:**
Alcoholic Liver Disease	ICD-9: 571.0, 571.1, 571.2, 571.3, 303, 305.0, 291, 357.5, 425.5, 535.3, 980.1, 980.9<br>ICD-10: K70, F10, E24.4, G62.1, I42.6, K29.2, G31.2, G72.1, K85.2, K86.0, T51.0, T51.9, Y57.3, X65, Z50.2, Z71.4, Z72.1
Viral Hepatitis	ICD-9: 070;<br>ICD-10: B16, B17, B18, B19
Biliary Disorders	ICD-9: 571.6, 576.1;<br>ICD-10: K83.0A, K83.0F, K74.3
Autoimmune Hepatitis	ICD-10: K75.4

Metabolic Disorders	ICD-9: 275.0 (Hemochromatosis), 275.1 (Wilson's disease), 277.6 (Other disorders of metabolism)<br><br>ICD-10: E83.1 (Disorders of iron metabolism), E83.02 (Wilson's disease), E88.01 (Alpha-1-antitrypsin deficiency), E88.02 (Other specified disorders of metabolism)
Vascular Disorders	ICD-9: 453.0 (Budd-Chiari syndrome)<br><br>ICD-10: I82.0 (Budd-Chiari syndrome)
Other Liver Disorders	ICD-9: 571.4 (Chronic hepatitis)<br><br>ICD-10: K76.5 (Hepatic veno-occlusive disease), K73.2, K73.9 (Chronic hepatitis), K74.4, K74.5 (Cirrhosis)

Abbreviations: DPP-4: dipeptidyl peptidase-4 inhibitors; GLP-1RAs: glucagon-like peptide-1 receptor agonists; SGLT-2is: sodium-glucose cotransporter-2 inhibitors; MASLD: Metabolic dysfunction-associated steatotic liver disease

Supplementary Table S3. Univariable and multivariable hazard ratios for MASLD based on demographic, socioeconomic, and metabolic variables

Category	Unadjusted HRs (95% CI)	Adjusted HR[1] (95% CI)
Food insecurity	1.98 (1.74–2.26)[b]	1.32 (1.13–1.53)[b]
Neighborhood deprivation	1.49 (1.31–1.69)[b]	1.20 (1.05–1.37)[b]
Age per decade	0.92 (0.89–0.95)[b]	0.89 (0.85–0.93)[b]
Sex: Male	-	-
Female	1.06 (0.94–1.19)	1.05 (0.93–1.19)
Race/ethnicity: Non-Hispanic White	-	-
Non-Hispanic Black	1.10 (0.90–1.35)	0.67 (0.54–0.83)[b]

Category	Unadjusted HRs (95% CI)	Adjusted HR[1] (95% CI)
Hispanic	1.78 (1.51–2.10)[b]	1.39 (1.16–1.68)[b]
Others (Non-Hispanic Asian, MENA, NHPI, AIAN)	1.01 (0.70–1.46)	1.08 (0.74–1.57)
Insurance category: None or public	-	-
Private insurance	0.90 (0.80–1.00)	1.05 (0.92–1.20)
Education: Less than HS/GED	-	-
HS/GED	0.82 (0.60–1.11)	1.02 (0.74–1.42)
Some College	0.77 (0.58–1.03)	1.05 (0.76–1.44)
College Degree	0.54 (0.40–0.72)[b]	0.91 (0.65–1.27)
Advanced Degree	0.41 (0.31–0.55)[b]	0.79 (0.56–1.11)
Income: < $10,000	-	-
$10–24,999	1.01 (0.80–1.29)	1.14 (0.89–1.46)
$25–34,999	0.76 (0.58–0.99)[a]	0.97 (0.73–1.30)
$35–49,999	0.78 (0.60–1.00)[a]	1.10 (0.84–1.45)
$50–74,999	0.71 (0.57–0.90)[b]	1.12 (0.86–1.47)
$75–99,999	0.61 (0.48–0.78)[b]	1.08 (0.81–1.44)
$100–149,999	0.54 (0.42–0.68)[b]	1.03 (0.77–1.38)
$150–199,999	0.47 (0.35–0.63)[b]	1.01 (0.71–1.42)
≥ $200,000	0.44 (0.33–0.58)[b]	1.05 (0.75–1.47)
Active Smoking	1.36 (1.12–1.65)[b]	1.04 (0.85–1.28)
Obesity	2.95 (2.63–3.32)[b]	2.29 (2.03–2.59)[b]
Hypertension	1.81 (1.62–2.03)[b]	1.43 (1.26–1.63)[b]
Type 2 diabetes	3.09 (2.72–3.52)[b]	1.89 (1.64–2.19)[b]

Category	Unadjusted HRs (95% CI)	Adjusted HR[1] (95% CI)
Hyperlipidemia	1.79 (1.60–2.00)[b]	1.50 (1.30–1.72)[b]

[a]$P < 0.05$. [b]$P < 0.01$.

[1]Multivariable hazard ratios are adjusted for food insecurity, age, sex, race/ethnicity, insurance category, education, income, active smoking, obesity, hypertension, type 2 diabetes, hyperlipidemia, and neighborhood deprivation.

Abbreviations: HS/GED: High School/General Educational Development; MASLD: Metabolic dysfunction-associated steatotic liver disease; MENA: Middle Eastern and North African; NHPI: Native Hawaiian and Other Pacific Islander; AIAN: American Indian and Alaska Native; HR: Hazard ratio

Supplementary Table S4. Sensitivity analyses for the association between food insecurity and incident MASLD

Analysis	Participants, *n*	Food Insecure, *n*	MASLD Events in FI, *n*	MASLD Events – FS, *n*	Adjusted HR (95% CI)	P value
Primary analysis	40,693	5494	292	948	1.32 (1.13–1.53)	<0.001
6-month lag	40,693	5494	292	948	1.34 (1.15–1.56)	<0.001
12-month lag	40,684	5490	288	943	1.32 (1.14–1.54)	<0.001
Strict MASLD definition	40,414	5439	134	435	1.44 (1.15–1.80)	0.002

Analysis	Participants, *n*	Food Insecure, *n*	MASLD Events in FI, *n*	MASLD Events – FS, *n*	Adjusted HR (95% CI)	P value
Propensity-score matched	10,720	5360	280	219	1.30 (1.09–1.55)	0.004
Stricter food insecurity	40,693	3252	179	1061	1.25 (1.04–1.49)	0.016
Birth-year adjusted	40,693	5494	292	948	1.33 (1.14–1.55)	<0.001
Alternative neighborhood deprivation	40,693	5494	292	948	1.34 (1.15–1.56)	<0.001

Abbreviations: FI: Food insecurity; FS: Food-secure; HR: Hazard ratio; CI: Confidence interval; MASLD: Metabolic dysfunction-associated steatotic liver disease

Supplementary Table S5. Sensitivity analyses for the association between neighborhood deprivation and incident MASLD

Analysis	Participants, *n*	Deprived Neighborhood, *n*	MASLD Events in Deprived, *n*	MASLD Events – Non-deprived, *n*	Adjusted HR (95% CI)	P value
Primary analysis	40,693	7633	319	921	1.20 (1.05–1.37)	0.008
6-month lag	40,693	7633	319	921	1.20 (1.05–1.37)	0.008

Analysis	Participants, *n*	Deprived Neighborhood, *n*	MASLD Events in Deprived, *n*	MASLD Events – Non-deprived, *n*	Adjusted HR (95% CI)	P value
12-month lag	40,684	7631	317	914	1.20 (1.05–1.37)	0.007
Strict MASLD definition	40,414	7631	126	443	1.19 (0.98–1.46)	0.079
Propensity-score matched	10,556	3813	203	295	1.26 (1.05–1.51)	0.012
Birth-year adjusted	40,693	7633	319	921	1.19 (1.04–1.36)	0.010

Abbreviations: HR: Hazard ratio; CI: Confidence interval; MASLD: Metabolic dysfunction-associated steatotic liver disease
